# A cotton MAPK cascade protects the cell wall against Verticillium wilt

**DOI:** 10.1093/plphys/kiag795

**Published:** 2026-10-23

**Authors:** Marcella Teixeira

**Affiliations:** Assistant Features Editor, Plant Physiology, American Society of Plant Biologists, Rockville, United States; Hermiston Agricultural Research and Extension Center, Department of Crop and Soil Science, Oregon State University, 2121 S 1st Street, Hermiston, OR 97838, United States

Plant immunity relies on defense responses that are conserved across species and effective against diverse pathogens. Once a pathogen is perceived at the cell surface, that signal must be relayed to the nucleus to reprogram gene expression. Mitogen-activated protein kinase (MAPK) cascades are central to this relay: a MAPK kinase kinase (MAPKKK) phosphorylates and activates a MAPK kinase (MKK), which in turn activates a MAPK that phosphorylates downstream targets, including transcription factors.

In Arabidopsis, the MAPKKK3/MAPKKK5-MKK4/MKK5-MPK3/MPK6 module has been extensively studied (Sun and Zhang 2022; Zhang and Zhang 2022). In wheat and sweet potato, MPK3 orthologs phosphorylate the transcription factors ERF3 and SPF1, respectively, changing downstream defense responses (Kim et al. 2019; Wang et al. 2021). MPK6 also connects immunity to hormone signaling, and in Arabidopsis, it interacts with and phosphorylates BRI1-EMS-SUPPRESSOR1 (BES1), a key transcription factor in brassinosteroid signaling (Kang et al. 2015).

Cotton (*Gossypium hirsutum*) is a pillar of the global textile industry, but its yield and fiber quality are threatened by several diseases, including Verticillium wilt, caused by *Verticillium dahliae* (Shaban et al. 2018; Zhu et al. 2023). Cotton MPK9 and MPK13 are induced by *V. dahliae* and contribute to resistance (Zhang et al. 2014; Mi et al. 2024), but how MPK13 transmits the defense signal remained unknown.

In this issue of *Plant Physiology*, Chen et al. (2026) show that GhMKK5 phosphorylates GhMPK13, which in turn phosphorylates GhBES1. Unphosphorylated GhBES1 binds the *GhPEL75* promoter and activates its expression. This is important, because GhPEL75 encodes a pectate lyase that degrades pectin and promotes susceptibility to *V. dahliae.* By phosphorylating GhBES1, GhMPK13 reduces *GhPEL75* expression, preserving cell wall pectin and enhancing resistance (Fig. 1).

**Figure 1:**
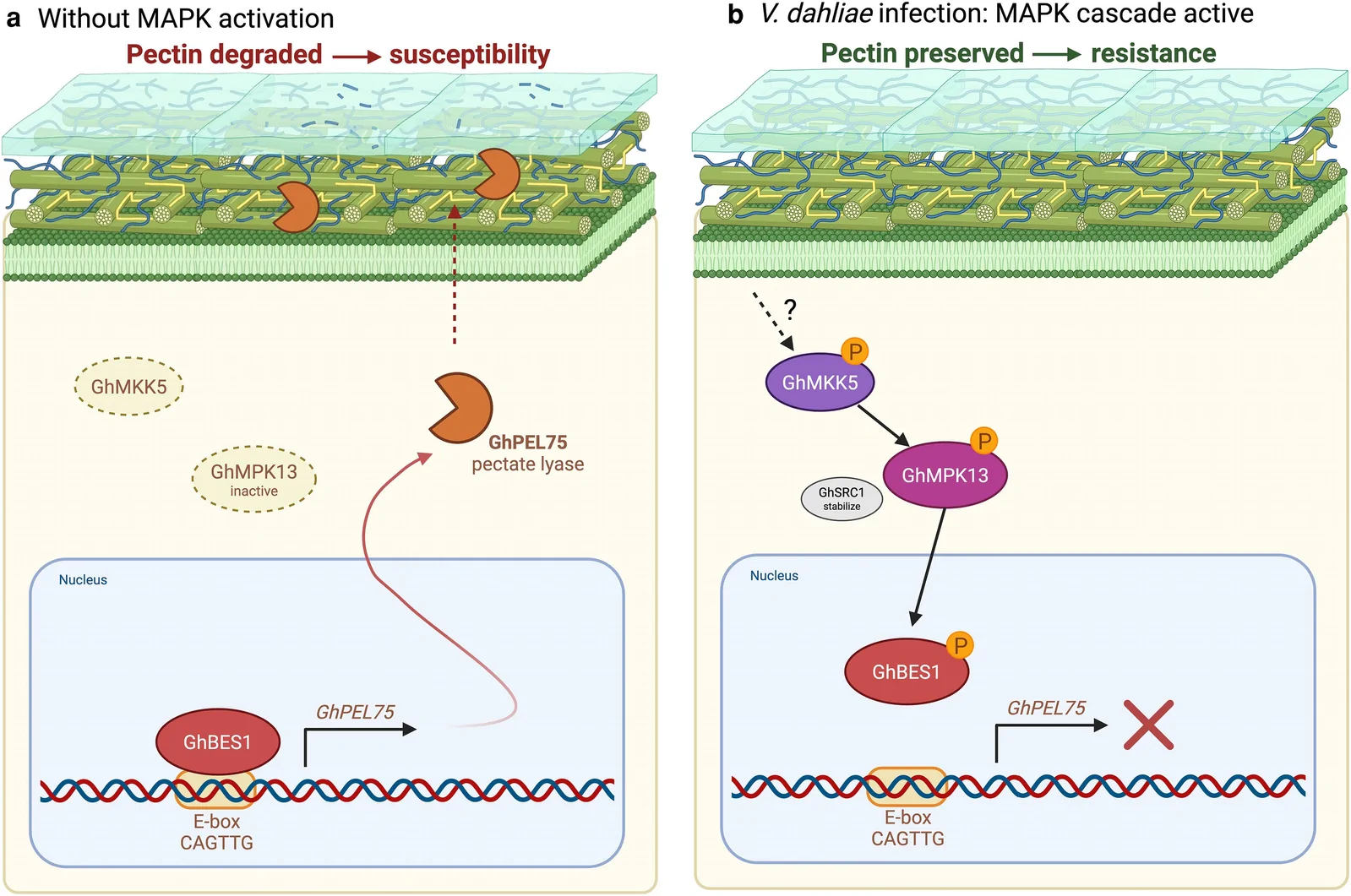
A MAPK cascade enhances cotton resistance to Verticillium wilt by inactivating a susceptibility module that degrades pectin. (a) In the absence of MAPK activation, GhMKK5 and GhMPK13 are inactive (dashed outlines). Unphosphorylated GhBES1 binds and E-box (CAGTTG) in the *GhPEL75* promoter and activates its transcription. The resulting pectate lyase, GhPEL75, degrades pectin in the cell wall, which facilitates penetration by *Verticillium dahliae* hyphae and leads to susceptibility. (b) During *V. dahliae* infection, GhMKK5 is activated by an unidentified upstream protein (?) and phosphorylates GhMPK13, which is stabilized by the dehydrin GhSRC1. GhMPK13 then phosphorylates GhBES1; Thr169 is proposed as the inactivating site. Phosphorylation reduces GhBES1 transcriptional activity and *GhPEL75* expression. Cell wall pectin is preserved, fungal penetration is restricted, and resistance increases. P, phosphorylation. Model based on Chen et al. (2026). Created in BioRender. Teixeira, M. (2026) https://BioRender.com/3oflsum.

To unravel this mechanism, the authors first confirmed GhMKK5 and GhMPK13 interaction using yeast two-hybrid (Y2H), pull-down with GhMKK5-GST and GhMPK13-His, and bimolecular fluorescence complementation (BiFC) with GhMKK5-cYFP and GhMPK13-nYFP. Additionally, colocalization using mCherry-GhMPK13 and GhMKK5-GFP showed that both proteins co-localized in *N. benthamiana* leaf epidermal cells. An in vitro kinase assay then revealed higher phosphorylation of GhMPK13 in the presence of GhMKK5.

What is the biological relevance of this phosphorylation? To answer this, the authors used virus-induced gene silencing (VIGS) to knockdown *GbMKK5* and *GbMPK13* in Hai7124, a resistant accession of *G. barbadense*. After inoculation with *V. dahliae,* both TRV:*GbMPK13* and TRV:*GbMKK5* displayed more severe symptoms, higher disease indices, and greater fungal biomass than controls, indicating that both genes positively regulate resistance. Because MAPK signaling depends on phosphorylation, the authors next treated leaves with chitin, a fungal molecular pattern recognized by plants, and probed MAPK phosphorylation with a phospho-p44/42 antibody. Chitin triggered strong GbMPK13 phosphorylation in control TRV:00 leaves, but this response was markedly reduced in both silenced lines.

To identify additional GhMPK13 partners, the authors performed Y2H using GhMPK13 as a bait, and recovered GhSRC1, a dehydrin. The interaction was confirmed by Y2H, pull-down, and BiFC, and the 2 proteins co-localized in *N. benthamiana* leaf epidermal cells.

GhSRC1 is highly expressed in roots and induced by *V. dahliae,* and silencing *GbSRC1* using VIGS increased symptoms, disease index and fungal biomass, indicating that it also promotes resistance in cotton. Because GhSRC1 lacks canonical MAPK phosphorylation sites and dehydrins are known to protect other proteins, the authors hypothesized it stabilizes GhMPK13. Indeed, GhMPK13 incubated in cotton protein extract was degraded less when GhSRC1 was present, and co-expression with GhSRC1 increased GhMPK13 accumulation in *N. benthamiana.*

To find GhMPK13 substrates, the authors compared the root transcriptomes of TRV:GbMPK13 and TRV:00 after inoculation with *V. dahliae.* They identified 100 genes that were repressed by *V. dahliae* in control plants, but upregulated when *GbMPK13* was silenced. Among them, there was a transcription factor with 11 putative MAPK phosphorylation sites, which the authors named GbBES1 based on its similarity to Arabidopsis BES1. Quantitative PCR confirmed that *GbBES1* expression was higher in TRV:*GbMPK13* plants than in controls after inoculation.

The interaction between GhMPK13 and GhBES1 was confirmed using Y2H, luciferase complementation imaging, and pull-down, and the 2 proteins co-localized in *N. benthamiana* leaf epidermal cells. An in vitro kinase assay showed that GhMPK13 strongly phosphorylates GhBES1, and mass spectrometry identified 4 phosphorylated residues: Ser164, Thr169, Ser200, and Ser224.

While *GbMPK13* silencing resulted in increased susceptibility to *V. dahliae*, silencing GhBES1 in the susceptible *G. hirsutum* accession TM-1 reduced symptoms, disease index and fungal biomass, indicating that GhBES1 negatively regulates resistance. This contrasts with Arabidopsis, where BES1 positively regulates immunity downstream of MPK6 (Kang et al. 2015), although BES1 has also been reported to suppress defense in *N. benthamiana* (Deng et al. 2016).

To identify GhBES1 targets, the authors performed a co-expression analysis and identified 105 genes co-expressed with *GbBES1*, enriched for pectin catabolism and including several pectate lyases. One of these, *GhPEL75,* carries a known BES1 binding site in its promoter, and its expression was higher in TRV: *GbMPK13* plants when compared to TRV:00 controls, suggesting GhBES1 activates *GhPEL75*.

Electrophoretic mobility shift and yeast one-hybrid assays showed that GhBES1 binds the *GhPEL75* promoter, and dual-luciferase reporter assays showed that it activates *GhPEL75* transcription. Consistently, silencing *GhBES1* reduced *GhPEL75* expression and pectate lyase activity in cotton. Notably, co-expressing GhMPK13 abolished GhBES1-driven reporter activity, indicating that GhMPK13 suppresses GhBES1 transcriptional activity.

Because GgMPK13 phosphorylates GhBES1, the authors asked whether GbBES1 phosphorylation interferes with its binding to *GhPEL75* promoter. They generated phospho-dead (alanine) and phospho-mimetic (glutamate) substitutions at each of the 4 sites, as well as quadruple phospho-dead (4A) and phospho-mimetic(4E) mutants, and tested them in dual-luciferase reporter assays. T169A mutant increased reporter activity, leading the authors to propose Thr169 as the residue through which GhMPK13 inactivates GhBES1.

Finally, the authors silenced GhPEL75. Twenty days after inoculation, TRV:*GhPEL75* plants showed lower symptoms, disease index, and fungal biomass than TRV:00 plants. They also had lower pectate lyase activity and higher pectin-derived galacturonic acid content, consistent with a more intact cell wall.

Together, these findings outline a pathway in which a MAPK cascade enhances resistance not by boosting defense hormone signaling, as is typical of MPK3/MPK6 modules, but by switching off a transcription factor that drives cell wall loosening. Several questions remain. The MAPKKK and receptor upstream of GhMKK5 are still unknown. In addition, because GhMKK5 and GhMPK13 were silenced in *G. barbadense* while GhBES1 and GhPEL75 were silenced in *G. hirsutum,* the full cascade has yet to be tested within a single genotype. The phosphorylation data also leaves some room for refinement: if phosphorylation inactivates GhBES1, phospho-dead mutants should be more active, yet only T169A behaved this way, while S164A and the 4A mutant were less active. Whether phosphorylation alters GhBES1 DNA binding, subcellular localization, or stability, and whether brassinosteroids modulate this module, remain open questions. Finally, because BES1 and pectin remodeling contribute to cell elongation, including cotton fiber development, it will be important to determine whether reducing GhBES1 or GhPEL75 activity to improve resistance comes at a cost to fiber quality. The answer will decide whether this pathway can be harnessed to breed cotton resistant to Verticillium wilt.

## Data Availability

No data are generated in this study.
